# *PPARGC1α* gene DNA methylation variations in human placenta mediate the link between maternal hyperglycemia and leptin levels in newborns

**DOI:** 10.1186/s13148-016-0239-9

**Published:** 2016-06-22

**Authors:** Sandra Côté, Valérie Gagné-Ouellet, Simon-Pierre Guay, Catherine Allard, Andrée-Anne Houde, Patrice Perron, Jean-Patrice Baillargeon, Daniel Gaudet, Renée Guérin, Diane Brisson, Marie-France Hivert, Luigi Bouchard

**Affiliations:** Department of Biochemistry, Université de Sherbrooke, Sherbrooke, QC Canada; ECOGENE-21 Laboratory and Lipid Clinic, CIUSSS du Saguenay-Lac-Saint-Jean - Hôpital de Chicoutimi, Saguenay, QC Canada; Department of Mathematics, Université de Sherbrooke, Sherbrooke, QC Canada; Department of Medicine, Université de Sherbrooke, Sherbrooke, QC Canada; Department of Medicine, Université de Montréal, Montréal, QC Canada; Department of Medical Biology, Chicoutimi Hospital, Saguenay, QC Canada; Department of Population Medicine, Harvard Pilgrim Health Care Institute, Harvard Medical School, Boston, MA USA

**Keywords:** Brown adipose tissue, Epigenetics, Gestational diabetes mellitus, Pregnancy, Hyperglycemia

## Abstract

**Background:**

Children exposed to gestational diabetes mellitus (GDM) are at a higher risk of developing obesity and type 2 diabetes. This susceptibility might involve brown adipose tissue (BAT), which is suspected to protect against obesity. The objective of this study is to assess whether fetal exposure to maternal hyperglycemia is associated with DNA methylation variations in genes involved in BAT genesis and activation.

**Methods:**

DNA methylation levels at the *PRDM16*, *BMP7*, *CTBP2*, and *PPARGC1α* gene loci were measured in placenta samples using bisulfite pyrosequencing in E-21 (*n* = 133; 33 cases of GDM) and the HumanMethylation450 array in Gen3G (*n* = 172, all from non-diabetic women) birth cohorts. Glucose tolerance was assessed in all women using an oral glucose tolerance test at the second trimester of pregnancy. Participating women were extensively phenotyped throughout pregnancy, and placenta and cord blood samples were collected at birth.

**Results:**

We report that maternal glycemia at the second and third trimester of pregnancy are correlated with variations in DNA methylation levels at *PRDM16*, *BMP7*, and *PPARGC1α* and with cord blood leptin levels. Variations in *PRDM16* and *PPARGC1α* DNA methylation levels were also correlated with cord blood leptin levels. Mediation analyses support that DNA methylation variations at the *PPARGC1α* gene locus explain 0.8 % of the cord blood leptin levels variance independently of maternal fasting glucose levels (*p* = 0.05).

**Conclusions:**

These results suggest that maternal glucose in pregnancy could produce variations in DNA methylation in BAT-related genes and that some of these DNA methylation marks seem to mediate the impact of maternal glycemia on cord blood leptin levels, an adipokine regulating body weight.

**Electronic supplementary material:**

The online version of this article (doi:10.1186/s13148-016-0239-9) contains supplementary material, which is available to authorized users.

## Background

The fetal metabolic programming and Developmental Origins of Health and Disease (DOHaD) hypotheses propose that the origin of chronic diseases such as obesity is related to an early exposure to a suboptimal fetal environment [1]. Indeed, Pedersen first suggested in 1954 that high concentrations of glucose in maternal diabetes give rise to a higher glucose transfer to the fetus and lead to fetal overgrowth [2]. The increased fetal growth is mainly a consequence of increased fetal insulin secretion (fetal hyperinsulinemia) acting on diverse tissues but with a stronger influence on adipose tissue development [3, 4]. Insulin does not cross the placental barrier. Nevertheless, the placenta has a key role in materno-fetal exchanges, nutrients partitioning (including glucose), and fetal energy metabolism and growth. The placenta undergoes various functional adaptations when exposed to metabolic challenges [5], which provides an opportunity to identify specific genes and pathways involved in placental energy transfer regulation, itself linked to an increased risk for childhood obesity [6, 7].

Gestational diabetes mellitus (GDM) is a form of diabetes first diagnosed in pregnancy, which now affects up to 16 % of pregnant women according to the International Association of the Diabetes and Pregnancy Study Groups (IADPSG) criteria [8]. GDM is diagnosed between 24 and 28 weeks of pregnancy, at the moment when maternal insulin resistance rises to derive the transfer of nutrients towards the fetus [9]. Human babies have the highest level of adiposity at birth compared to other species, and most of the fat accretion also occurs in the second half of pregnancy. After birth, newborns’ brown adipose tissue (BAT) and beige adipocytes (brown-like adipocytes within white adipose tissue (wBAT)) are essential to maintain body temperature [10]. Until recently, BAT was believed to be lacking at adulthood [11]. However, BAT has now been observed in adults and found metabolically active following a cold-exposure, leading to an increase of total energy expenditure [12]. In fact, the lack of thermogenic activation has been associated with obesity in previous animal and human studies [10, 13–17]. This has re-established the potential role of BAT in energy balance regulation in adults and suggests that the dysregulation of BAT might be involved in the development of obesity and its associated metabolic disorders [15]. wBAT is believed to have a role and importance similar to those of BAT, but its contribution to the energy balance in humans is not yet well understood [18]. Many gestational events might influence fetal adipogenesis, including BAT and wBAT development [19], but the specific impacts of these events need to be better defined.

Few genes are central to the BAT/wBAT genesis regulation. The main and necessary regulator is the PR domain-containing protein 16 (*PRDM16*) transcription factor that triggers the activation of the brown adipogenic program. Through its interaction with the C-terminal binding protein 2 (*CTBP2*) transcriptional co-repressor, *PRDM16* blocks the induction of the genes required for muscle cell or white adipocyte differentiation from myogenic factor 5-positive (*MYF5+*) (precursor brown adipocytes) or MYF5-negative (*MYF5−*) cells (likely precursors of beige adipocytes) [1, 20]. Peroxisome proliferator-activated receptor-gamma, co-activator 1, alpha (*PPARGC1α*) is a transcriptional co-activator required for mitochondrial biogenesis and thermogenesis induction through adrenergic activation in both brown and beige adipocytes [21, 22]. PPARGC1α participates but is not essential for normal BAT/wBAT genesis [23]; however, PPARGC1α is also involved in the glycemic/lipid metabolism in many other tissues, including the muscles and liver. Finally, the bone morphogenetic protein 7 (*BMP7*) is essential to BAT/wBAT genesis as it induces the expression of the *PRDM16* and *PPARGC1α* genes [24].

Although the molecular mechanisms involved in fetal metabolic programming remain unclear, it is strongly suggested that epigenetic mechanisms are central to this phenomena [25]. Epigenetic mechanisms, including DNA methylation, are involved in cellular differentiation in the BAT/wBAT genesis [26–28]. In the current study, we tested in two independent birth cohorts whether the fetal exposure to maternal hyperglycemia is associated with placental DNA methylation variations in genes involved in BAT/wBAT genesis, specifically targeting the *PRDM16*, *BMP7*, *CTBP2*, and *PPARGC1α* gene loci. We assessed whether the selected epivariations mediated the link between maternal glycemia and adiposity markers in newborns.

## Methods

### Subjects

#### E-21 birth cohort

The ECOGENE-21 (E-21) birth cohort initially recruited 241 Caucasian women during their first trimester of pregnancy in the Saguenay city area, Quebec, Canada, from 2006 to 2010. The inclusion criteria were as follows: 18 < age < 40 years and between the 12th and 14th week of pregnancy. The exclusion criteria were as follows: glycemia perturbation diagnosed before pregnancy, current multiple pregnancy, positive history of alcohol and/or drug abuse during the current pregnancy, severe mental illness or mental retardation, uncontrolled thyroid disorders, renal insufficiency and use of drugs affecting plasma glucose and lipid levels. The Chicoutimi Hospital Ethics Committee approved the project. All women provided a written informed consent before their inclusion in the study, in accordance with the Declaration of Helsinki.

All women were invited for research visits under fasting state at the E-21 Research Centre (Chicoutimi, Saguenay) at the end of each trimester of pregnancy. BMI was measured according to the standardized procedures of the Airlie conference [29]. Blood glucose measurements were made on fresh serum samples at the Chicoutimi Hospital Clinical Laboratory. Glucose was evaluated using a Beckman analyser (model CX 7; Beckman, Coulter, USA) as recommended by the manufacturer. Glucose tolerance was assessed using a 75-g oral glucose tolerance test (OGTT) performed at 24–28 weeks’ gestation. Women with a 2-h post-OGTT glucose level ≥7.8 mmol/L were classified as GDM (*n* = 55) according to the World Health Organization (WHO) criteria [30]. They were treated with diet only (*n* = 28) or with diet and insulin (*n* = 27). The homeostasis model assessment of insulin resistance (HOMA-IR) was calculated according to the validated formula: HOMA-IR = (fasting insulin × fasting glucose)/22.5 [31]. At delivery, metabolic and anthropometric measures of newborns were taken. Leptin levels were measured in the cord blood using ELISA following manufacturer instructions (B-Bridge International, USA).

Analyses were carried out using 133 participants out of the 241; indeed, we only selected the mother and child pairs with available placenta samples and complete phenotypic information related to maternal glycemia and gestational diabetes. The selected participants did not differ in age and BMI at the first trimester of pregnancy, suggesting that no selection bias was introduced.

#### Genetics of Glucose regulation in Gestation and Growth (Gen3G)

We recruited pregnant women at the first trimester (5 to 16 weeks) of pregnancy in the Sherbrooke city area, Quebec, Canada, from 2010 to 2013. We excluded women who had any of the following criteria: age <18 or >40 years, multiple pregnancy, pre-gestational diabetes or diabetes discovered at the first trimester of pregnancy, or other major medical conditions that would affect glucose regulation. The ethical review board from the *Centre hospitalier universitaire de Sherbrooke* approved the project, and all women gave a written informed consent before they were included in the study, in accordance with the Declaration of Helsinki.

During the first visit, the baseline characteristics of participants were collected. Anthropometry was measured according to the standardized procedures, and information on lifestyle was collected using validated questionnaires [32, 33]. At the second trimester, in addition to anthropometric measures and repeated lifestyle questionnaires, a 75-g OGTT under the fasting state was performed for all women. Women were classified as GDM according to the IADPSG criteria (fasting ≥5.1 mmol/L; 1 h ≥10.0 mmol/L; and 2 h ≥8.5 mmol/L). Extra blood samples were collected in the fasted state as well as at 1 and 2 h during the OGTT. At delivery, clinical information about all obstetrical and neonatal medical events was collected. A trained research staff collected the cord blood and placenta biopsies according to the standardized procedures described below.

### Placenta sampling

Placenta samples from the E-21 and Gen3G cohorts were collected on average <15 min after delivery by a well-trained staff and kept in RNALater (Qiagen, USA) at −80 °C until nucleic acid extraction. Tissue biopsies were collected near the insertion of the umbilical cord (within 2 cm) from the fetal side and consisted of a mix of chorionic villi mainly composed of trophoblast cells (cyto and syncytiotrophoblast).

### DNA extraction

DNA was purified from fetal placenta biopsies using the All Prep DNA/RNA/Protein Mini Kit (Qiagen, USA). DNA purity was assessed using the ratio of absorbance at 260 to 280 nm with the Ultrospec 2000 UV/Visible Spectrophotometer (Pharmacia Biotech, USA) [34].

### DNA methylation quantification in placenta

#### E-21 birth cohort

The CpG sites were selected based on our previous epigenome-wide association study (EWAS) as well as from the literature [35–38]. The accurate and quantitative pyrosequencing technology was used to determine base-specific cytosine methylation levels at four gene loci: *PRDM16*, *BMP7*, *CTBP2*, and *PPARGC1α*. DNA was first treated with sodium bisulfite (EpiTech Bisulfite Kit; Qiagen, USA), amplified by PCR (Pyromark PCR Kit; Qiagen, USA) and sequenced by synthesis assay (Pyromark Gold Q24 Reagents; Qiagen, USA) of the target sequence. PCR primers were selected using the PyroMark Assay Design software (v2.0.1.15) of Qiagen (USA). Overall, four CpGs were epigenotyped at the *PRDM16* locus, four at the *BMP7* locus, six at the *CTBP2* locus, and four at the *PPPARGC1α* locus. The four CPGs of *BMP7*, six CpGs of *CTBP2*, and *PPARGC1α* CpG 1 and 2 DNA methylation levels were highly correlated within their respective genomic region (*r* > 0.80; *p* < 0.05) (Additional file 1: Figure S1). Statistical analyses were thus performed with these CpGs, on mean DNA methylation values. The PCR and pyrosequencing primers for all the CpGs tested within the *PRDM16*, *BMP7*, *CTBP2*, and *PPARGC1α* gene loci are provided in Additional file 2: Table S1. Details on bis-pyrosequencing quality control are provided in [39]. Results were excluded and reanalysed when they failed to pass quality controls as recommended by the manufacturers.

#### Gen3G birth cohort

We performed an in silico replication study in Gen3G [33] using data available from HumanMethylation450 BeadChip Arrays (Illumina Inc, USA) performed in 172 placental samples randomly selected from non-diabetic women. Briefly, the HumanMethylation450 array allows the quantification of 485,764 specific CpG sites within the whole genome, covering 21,231 genes. It has been shown that this array is accurate, reproducible, and congruent with bis-pyrosequencing data [40]. If not covered, CpG probes in close vicinity were selected such for *PRDM16* cg06814194 and cg23738647, *BMP7* cg18759209, and *PPARGC1α* cg11270806 and cg27514608. This analytical strategy (in silico replication of significant CpGs identified in E-21) allowed us to reduce the number of statistical tests in the replication cohort and provides independent samples for results’ validation [41–43]. DNA methylation levels at the *PRDM16*, *BMP7*, *CTBP2*, and *PPARGC1α* genes loci (by Illumina annotation) were extracted, and data were rank-normalized before statistical analyses (see section below).

### Statistical analyses

#### E-21 birth cohort

We compared groups (normoglycemic (NGT) vs. GDM)) using the Pearson *χ*^2^-statistic for categorical variables and Wilcoxon-Mann-Whitney’s rank-sum test for continuous variables. Because data were not normally distributed, Spearman’s rank correlation was used to assess the association between maternal variables (fasting glucose and insulin levels, HOMA-IR at the second and third trimester of pregnancy, and 2 h post-OGTT glucose levels as diagnostic criteria for GDM) and cord blood leptin and glucose levels. We assessed associations between fetal placenta DNA methylation levels and maternal variables. The maternal variables used were the same as above. We further pursued our investigations by testing associations between fetal placenta DNA methylation, maternal glucose levels, cord blood leptin levels, and newborn’s anthropometric measurements (weight, height, head circumference, and chest circumference) as proxy for the downstream effect of the epigenetic modulation of BAT-related loci. Residual scores of DNA methylation levels and cord blood leptin and glucose concentrations were used in all statistical models. Residual scores of DNA methylation levels were obtained by using the unstandardized residuals computed by linear regressions which included co-variables known to increase the risk of GDM or influence fetal growth: gestational age, newborn’s sex, birth weight, smoking during pregnancy, weight gain between the first and second trimester, and 2 h post-OGTT glucose concentration, maternal age, and BMI at the first trimester. Residual scores of cord blood leptin and glucose levels were obtained considering gestational age, newborn’s sex, and birth weight. Covariates were selected based on their role in the pathophysiology of maternal hyperglycemia and GDM and/or previously associated with glucose homeostasis dysregulation.

Stepwise multivariable linear regression was also used to identify predictors of cord blood leptin levels. The input-independent variables were as follows: DNA methylation levels of all loci investigated in this study, gestational age, newborns’ sex, smoking during pregnancy, maternal BMI at the first trimester, weight gain between the first and second trimester, and 2 h post-OGTT glucose levels. Only variables with a *p* < 0.05 were included in the final regression model.

We estimated the direct association between maternal and fetal metabolic conditions and the indirect effect through DNA methylation (mediator) using a non-parametric bias-corrected bootstrapping procedure providing an empirical estimation of the distribution of the samples using 5000 resampling [44]. This allowed us to assess whether the selected epivariations mediate the association (or support a causality link) between maternal glycemia and offspring leptin levels. As above, confounders and covariates (gestational age, newborn’s sex, birth weight, smoking during pregnancy, weight gain between the first and second trimester and 2 h post-OGTT glucose concentration, maternal age, and BMI at the first trimester) were considered in the statistical models. Mediation analyses were conducted using the INDIRECT macro as implemented in the SPSS statistical software (version 22.0.0) (IBM, USA) [44]. Results were considered significant if *p* < 0.05 (two-sided). All statistical analyses were performed using the SPSS software.

#### Gen3G birth cohort

All probes that reached *p* < 0.05 for association between maternal glycemia or GDM exposure and DNA methylation levels in E-21 were taken for an in silico replication in Gen3G. If a probe was not directly available on the 450k arrays, then we used proxy probes selected for their genomic proximity to the associated CpGs. We used Spearman’s rank correlations to assess the association between fetal placenta DNA methylation levels and maternal variables as prenatal exposures of interest: fasting glucose and insulin levels, 2 h post-OGTT glucose levels, and HOMA-IR. We also assessed correlations between fetal DNA methylation and cord blood leptin and glucose levels using Spearman’s. Residual scores of DNA methylation levels were used in all the statistical models that included this variable. Residual scores of DNA methylation levels were obtained by the same method as with E-21. The sample sizes for some analyses were lower than 172 since some participants had missing co-variables (167–170). Results were considered statistically significant when *p* < 0.05 (two-sided). All analyses were performed using the R software (v3.0.2).

## Results

### Clinical characteristics of the mothers and newborns

Table 1 presents the characteristics of the 133 women and newborn pairs from E-21 according to the GDM status and from the 172 dyads from Gen3G. In E-21, women with GDM (*n* = 33) had very similar anthropometric and metabolic profiles on average when compared to the NGT women group, including BMI at the first trimester of pregnancy (25.9 vs. 25.1; *p* = 0.16). Age and BMI at the first trimester of pregnancy were similar in both cohorts (Table 1).

**Table 1:** Characteristics of mothers and newborns in the E-21 and Gen3G birth cohorts

Characteristics	E-21—NGT	E-21—GDM^a^		Gen3G	
	*n* = 100	*n* = 33	*p*	*n* = 172	*p* ^p^
Maternal characteristics					
Gravidity					
0	38	6	0.88	60	0.79
1	32	6		60	
2	19	4		32	
3	4	0		13	
4+	3	1		7	
Parity					
0	49	7	0.62	88	0.32
1	30	8		60	
2	16	2		18	
3+	1	0		6	
Smoking during pregnancy (%)	9.0	0	0.074	7.1^l^	0.73
Mode of delivery (*n*)					
C-section	15	4	0.36^c^	30	0.83
Vaginal	81	12		142	
1st trimester of pregnancy					
Age (years)	28.6 ± 3.9	29.2 ± 3.8	0.40	28.0 ± 4.1	0.26
BMI (kg/m^2^)	25.1 ± 5.0	25.9 ± 4.0	0.16	25.6 ± 5.6	0.71
Glucose (mmol/L)	4.44 ± 0.34^b^	4.49 ± 0.50^b^	0.53	5.31 ± 1.27^m, n^	<0.001
2nd trimester of pregnancy					
Weight gain between 1st and 2nd trimester (%)	10.0 ± 4.4	9.0 ± 2.7	0.27	10.3 ± 5.1	0.36
Fasting glucose (mmol/L)	4.31 ± 0.39^b^	4.54 ± 0.45^b^	0.006^d^	4.11 ± 0.32	<0.001
Fasting insulin (mU/L)	9.53 ± 8.71	11.02 ± 6.00	0.048^e^	8.64 ± 5.33^l^	0.81
2 h post-OGTT glucose concentration (mmol/L)	6.19 ± 0.81	8.48 ± 0.67	<0.001	5.69 ± 1.08^n^	<0.001
HOMA-IR	1.83 ± 1.57	2.15 ± 1.29	0.30	1.60 ± 1.02	0.42
3rd trimester of pregnancy					
Weight gain between 2nd and 3rd trimester (%)	12.54 ± 3.97	9.20 ± 3.44	<0.001	–	–
Fasting glucose (mmol/L)	4.32 ± 0.73^b^	4.25 ± 0.43^b^	0.60	–	–
Fasting insulin (mU/L)	14.84 ± 26.50	9.43 ± 5.31	0.33	–	–
HOMA-IR^a^	3.41 ± 9.81	1.81 ± 1.09	0.13	–	–
Newborns’ characteristics					
Gestational age (week)	39.4 ± 1.0	39.2 ± 1.5	0.62	39.5 ± 1.2	0.20
Sex (boys/girls)	50/50	18/15	0.65	81/91	0.74
Birth weight (kg)	3.46 ± 0.37	3.40 ± 0.46	0.33	3.43 ± 0.44	0.38
Chest circumference (cm)	33.47 ± 1.61	33.07 ± 1.85	0.21^f^	–	–
Head circumference (cm)	34.19 ± 1.49	34.63 ± 1.22	0.12^g^	34.62 ± 1.42	0.05
Birth height (cm)	49.87 ± 1.91	49.82 ± 1.56	0.79^h^	50.86 ± 2.07^n^	<0.001
Cord blood leptin (ng/mL)	11.1 ± 8.39	7.55 ± 4.70	0.10^i^	15.0 ± 14.1^o^	0.13
Cord blood glucose (mmol/L)	4.55 ± 1.01	4.60 ± 0.78	0.64^j^	4.37 ± 0.91^l^	0.17
Cord blood triglycerides (mmol/L)	0.42 ± 0.15	0.51 ± 0.28	0.52^k^	–	–

Continuous variables are mean +/−s.d

^a^Treated GDM with diet or with insulin and diet

^b^Fasting state

^c^ *n* = 112 (96 NGT and 16 GDM)

^d^ *n* = 130 (100 NGT and 30 GDM)

^e^ *n* = 125 (97 NGT and 28 GDM)

^f^ *n* = 122 (97 NGT and 25 GDM)

^g^ *n* = 105 (77 NGT and 28 GDM)

^h^ *n* = 123 (93 NGT and 30 GDM)

^i^ *n* = 123 (94 NGT and 30 GDM)

^j^ *n* = 78 (57 NGT and 21 GDM)

^k^ *n* = 116 (86 NGT and 30 GDM)

^l^ *n* = 170

^m^1 h-post 50 g OGTT

^n^ *n* = 171

^o^ *n* = 169

^p^Comparison between NGT patients in E-21 and Gen3G

**Fig. 1:**
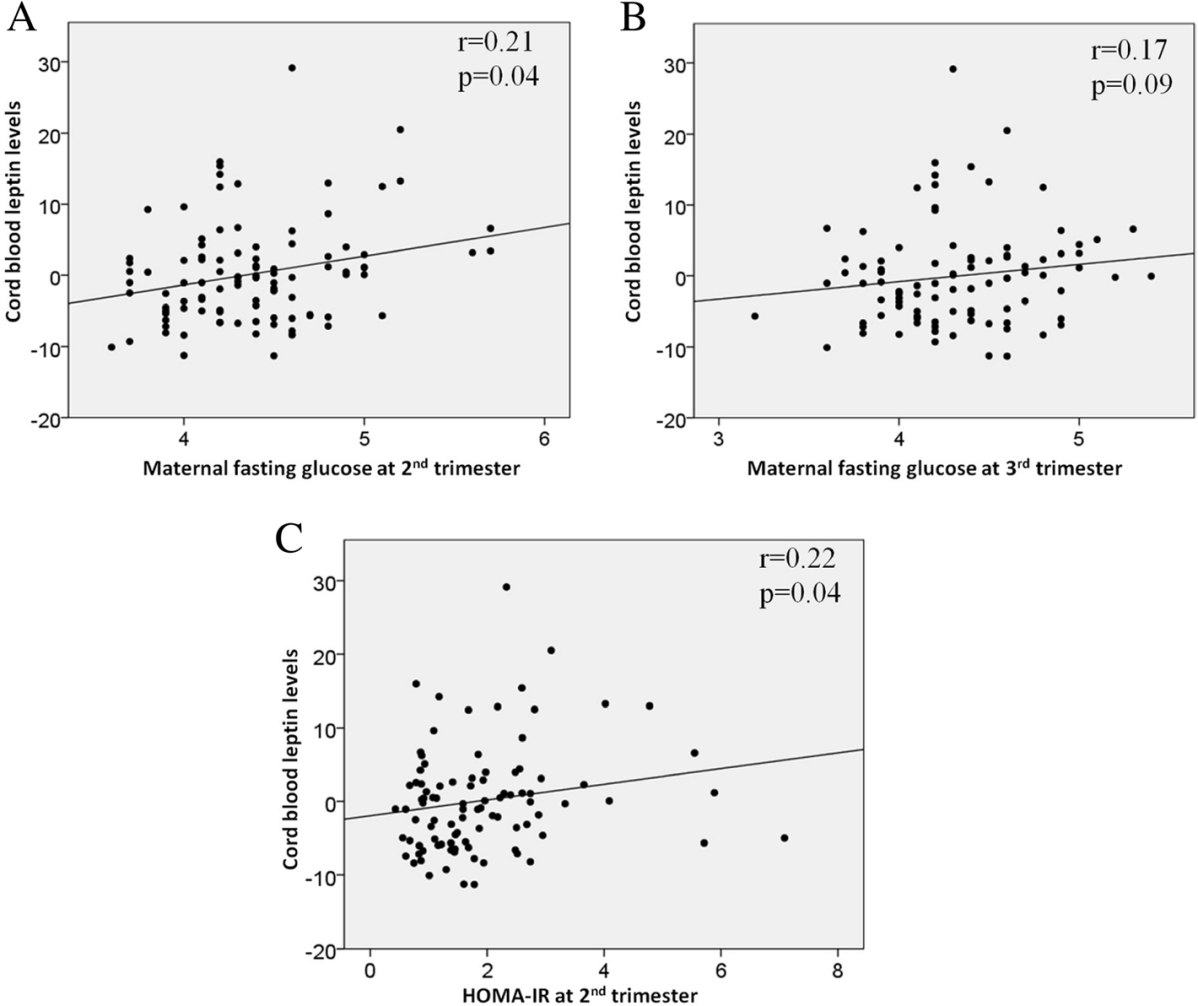
Maternal glycemia correlations with cord blood leptin levels. Maternal glycemia was correlated with cord blood leptin levels. **a** Fasting glucose at second trimester; **b** third trimester of pregnancy and **c** HOMA-IR at the second trimester of pregnancy were correlated with cord blood leptin levels. Unstandardized residuals were first computed for DNA methylation levels using linear regression and gestational age, newborn’s sex and weight, smoking during pregnancy, weight gain between the first and second trimester of pregnancy and maternal BMI at the first trimester of pregnancy as covariates. The residuals were then used to compute Spearman’s correlation coefficients

We first investigated whether maternal glucose-related phenotypes in pregnancy were associated with the newborn’s adiposity and metabolic markers. In the whole E-21 cohort, a higher maternal glycemia was associated with higher cord blood leptin levels (second trimester *r* = 0.21, *p* = 0.04; third trimester *r* = 0.18, *p* = 0.08) (Fig. 1a, b). A higher HOMA-IR at the second trimester of pregnancy was also significantly associated with higher cord blood leptin levels (*r* = 0.22, *p* = 0.04) (Fig. 1c). We also tested associations between maternal glycemia at the second and third trimester of pregnancy and cord blood glucose levels, but neither was significant (*r* = 0.12, *p* = 0.32 and *r* = −0.15, *p* = 0.20, respectively).

### Association between DNA methylation and maternal hyperglycemia (GDM)

We assessed in E-21 whether the maternal glycemic profile at the second and third trimester of pregnancy was associated with variations in placental DNA methylation levels (Table 2). At the *PPARGC1α* gene locus, we observed that a higher DNA methylation levels were associated with higher second trimester fasting glucose levels (CpG3, *r* = 0.18, *p* = 0.05 and CpG4, *r* = 0.23, *p* = 0.008) and 2 h post-OGTT glycemia (CpG4, *r* = 0.22, *p* = 0.01) (Table 2). We also observed that lower *PRDM16* DNA methylation levels were associated with higher fasting glucose levels at the second (CpG3, *r* = −0.18, *p* = 0.04) and third trimester (CpG2, *r* = −0.24, *p* = 0.007). Lower *BMP7* DNA methylation levels were associated with a higher 2 h post-OGTT glycemia (mean methylation; *r* = −0.18, *p* = 0.04) (Table 2).

**Table 2:** Spearman’s correlation coefficients between placental DNA methylation and metabolic variables of mothers (second and third trimester of pregnancy)—E-21 birth cohort

	2nd trimester				3rd trimester		
DNA methylation levels^a^	Fasting glucose levels	Fasting insulin levels	2 h post-OGTT glucose levels	HOMA-IR	Fasting glucose levels	Fasting insulin levels	HOMA-IR
	*n* = 130	*n* = 125	*n* = 133	*n* = 122	*n* = 133	*n* = 133	*N* = 116
*PRDM16*-CpG1	r = −0.04	**r =** −**0.18**	r = −0.05	**r =** −**0.17**	r = 0.02	r = −0.09	r = −0.088
	NS	**p** ***=*** **0.05**	NS	**p** ***=*** **0.05**	NS	NS	NS
*PRDM16*-CpG2	r = −0.14	r = −0.13	r = −0.04	*r =* −*0.15*	**r = 0.24**	r = 0.101	*r = 0.162*
	p *=* 0.12	p = 0.14	NS	*p = 0.10*	**p = 0.007**	NS	*p = 0.08*
*PRDM16*-CpG3	**r =** −**0.18**	r = −0.05	r = −0.04	r = −0.08	r = 0.10	0.13	r = 0.106
	**p** ***=*** **0.04**	NS	NS	NS	NS	NS	NS
*PRDM16*-CpG4	r = −0.13	r = −0.04	r = −0.06	r = −0.06	r = 0.09	r = 0.03	r = 0.042
	p = 0.15	NS	NS	NS	NS	NS	NS
Mean *BMP7*	r = −0.09	r = −0.09	**r =** −**0.18**	r = −0.07	r = 0.09	r = 0.05	r = 0.007
	NS	NS	**p = 0.04**	NS	NS	NS	NS
Mean *CTBP2*	*r =* −*0.15*	r = −0.13	r = −0.02	*r =* −*0.17*	r = 0.06	r = 0.08	r = 0.056
	*p = 0.10*	p = 0.15	NS	*p = 0.06*	NS	NS	NS
Mean *PPARGC1α*-CpG1,2	r = −0.29	r = −0.13	r = −0.05	r = −0.13	r = −0.05	**r = 0.41**	r = −0.033
	NS	p = 0.15	NS	p = 0.18	NS	**p** ***<*** **0.001**	NS
*PPARGC1α*-CpG3	**r = 0.18**	r = −0.01	*r = 0.16*	r = 0.02	r = 0.067	r = 0.06	r = 0.073
	**p = 0.05**	NS	*p = 0.07*	NS	NS	NS	NS
*PPARGC1α*-CpG4	**r = 0.23**	r = 0.13	**r = 0.22**	*r = 0.17*	r = 0.109	r = 0.032	=0.018
	**p = 0.008**	p = 0.14	**p = 0.01**	*p = 0.08*	NS	NS	NS

^a^Residual scores of DNA methylation levels were used in the statistical models. They were obtained by using unstandardized analysis of residuals computed by linear regressions which included gestational age, newborn’s sex and weight, smoking during pregnancy, weight gain between the first and second trimester and maternal BMI at the first trimester. Statistically significant results (*p* ≤ 0.05) are shown in bold, whereas statistical trends (*p* ≤ 0.10) are shown in italics. NS = *p >* 0.15

In concordance with the analyses of the continuous glycemic traits, *BMP7* and *PRDM16* DNA methylation levels were lower in placenta exposed to GDM as compared to those not exposed (*BMP7* mean: 27.4 ± 4.8 vs. 29.9 ± 6.9; *p* = 0.03 and *PRDM16* - CpG3; 38.8 ± 15.6 vs. 43.8 ± 13.7, *p* = 0.08) (Table 3). Similarly, DNA methylation levels at *PPARGC1α* tended to be higher in placenta exposed to GDM (CpG3; 49.8 ± 13.9 vs. 46.7 ± 15.0, *p* = 0.17 and CpG4; 55.2 ± 13.5 vs. 51.0 ± 12.5, *p* = 0.09). Although GDM exposure results are in the same direction of effect as that of the correlations with continuous maternal glycemic traits in E-21, only the comparison at *BMP7* reached statistical significance levels (Table 3).

**Table 3:** Placental DNA methylation levels (%) according to maternal GDM—E-21 birth cohort

DNA methylation levels^a^	NGT mother	GDM mother	*p*
	*n* = 100	*n* = 33	
*PRDM16*-CpG1	29.4 ± 14.1	26.9 ± 9.2	0.93
*PRDM16*-CpG2	33.7 ± 13.9	29.2 ± 14.7	0.12
*PRDM16*-CpG3	43.8 ± 13.7	38.8 ± 15.6	*0.08*
*PRDM16*-CpG4	23.5 ± 10.4	22.2 ± 13.0	0.24
Mean *BMP7*	29.9 ± 6.9	27.4 ± 4.8	**0.03**
Mean *CTBP2*	13.8 ± 1.4	13.7 ± 2.4	0.54
Mean*PPARGC1α-*CpG1,2^b, c^	12.1 ± 8.2	10.4 ± 5.7	0.78
*PPARGC1α*-CpG3	46.7 ± 15.0	49.8 ± 13.9	0.17
*PPARGC1α*-CpG4	51.0 ± 12.5	55.2 ± 13.5	*0.09*

^a^Residual scores of DNA methylation levels were used in the statistical models. They were obtained by using unstandardized analysis of residuals computed by linear regressions which included gestational age, newborn’s sex and weight, smoking during pregnancy, weight gain between the first and second trimester and maternal BMI at the first trimester

^b^ *n* = 129 (70 normal weight and 59 overweight)

^c^ *n* = 129 (96 NGTs and 33 GDMs). Statistically significant results (*p* < 0.05) are shown in bold, whereas statistical trend (*p* < 0.10) are shown in italics

We then conducted in silico replications in placental samples from 172 NGT mothers from Gen3G (Table 4). First, we confirmed that higher DNA methylation levels at *PPARGC1α* were associated with a higher glucose 2 h post-OGTT (cg27514608, *r* = 0.20, *p* = 0.008 and cg11270806, *r* = 0.27, *p* < 0.001). In addition, lower levels of DNA methylation at *PRDM16* (cg04873098 probe corresponding to CpG2, *r* = −0.14, *p* = 0.07) and *BMP7* (cg18759209 probe corresponding to CpG1, *r* = −0.16, *p* = 0.04) were associated with higher maternal fasting glucose levels at the second trimester. This result also confirms the direction of the correlations between maternal hyperglycemia and the DNA methylation profile at these two loci, as was observed in E-21.

**Table 4:** Spearman’s correlation coefficients between placental DNA methylation and metabolic variables of the mothers (second trimester of pregnancy)—Gen3G birth cohort

DNA methylation levels^a^	Maternal fasting glucose levels	Maternal fasting insulin levels	2 h post-OGTT glucose levels
	*n* = 170	*n* = 168	*n* = 169
*PRDM16-*CpG1 cg01046951	r = 0.005	**r = 0.16**	r = −0.05
	NS	**p = 0.04**	NS
*PRDM16-*CpG2 cg04873098	*r =* −*0.14*	r = 0.02	r = 0.03
	*p = 0.07*	NS	NS
*PRDM16* cg06814194	r = −0.02	r = 0.10	r = −0.07
	NS	NS	NS
*PRDM16* cg23738647	r = 0.02	r = 0.08	r = −0.08
	NS	NS	NS
*BMP7* cg18759209	**r =** −**0.16**	r = −0.06	r = −0.10
	**p = 0.04**	NS	NS
*PPARGC1α* cg11270806	r = 0.005	r = 0.09	**r = 0.27**
	NS	NS	**p < 0.001**
*PPARGC1α* cg27514608	r = 0.03	r = 0.12	**r = 0.20**
	NS	NS	**p = 0.008**
*PPARGC1α-*CpG3 cg08550435	r = 0.04	r = 0.08	r = −0.006
	NS	NS	NS

^a^Residual scores of DNA methylation levels were used in the statistical models. They were obtained by using unstandardized analysis of residuals computed by linear regressions which included gestational age, newborn’s sex and weight, smoking during pregnancy, weight gain between the first and second trimester and maternal BMI at the first trimester. Statistically significant results (*p* ≤ 0.05) are shown in bold, whereas statistical trends (*p* ≤ 0.10) are shown in italics. NS = *p* > 0.15

### DNA methylation levels in newborns: association with adiposity and metabolic markers

**Fig. 2:**
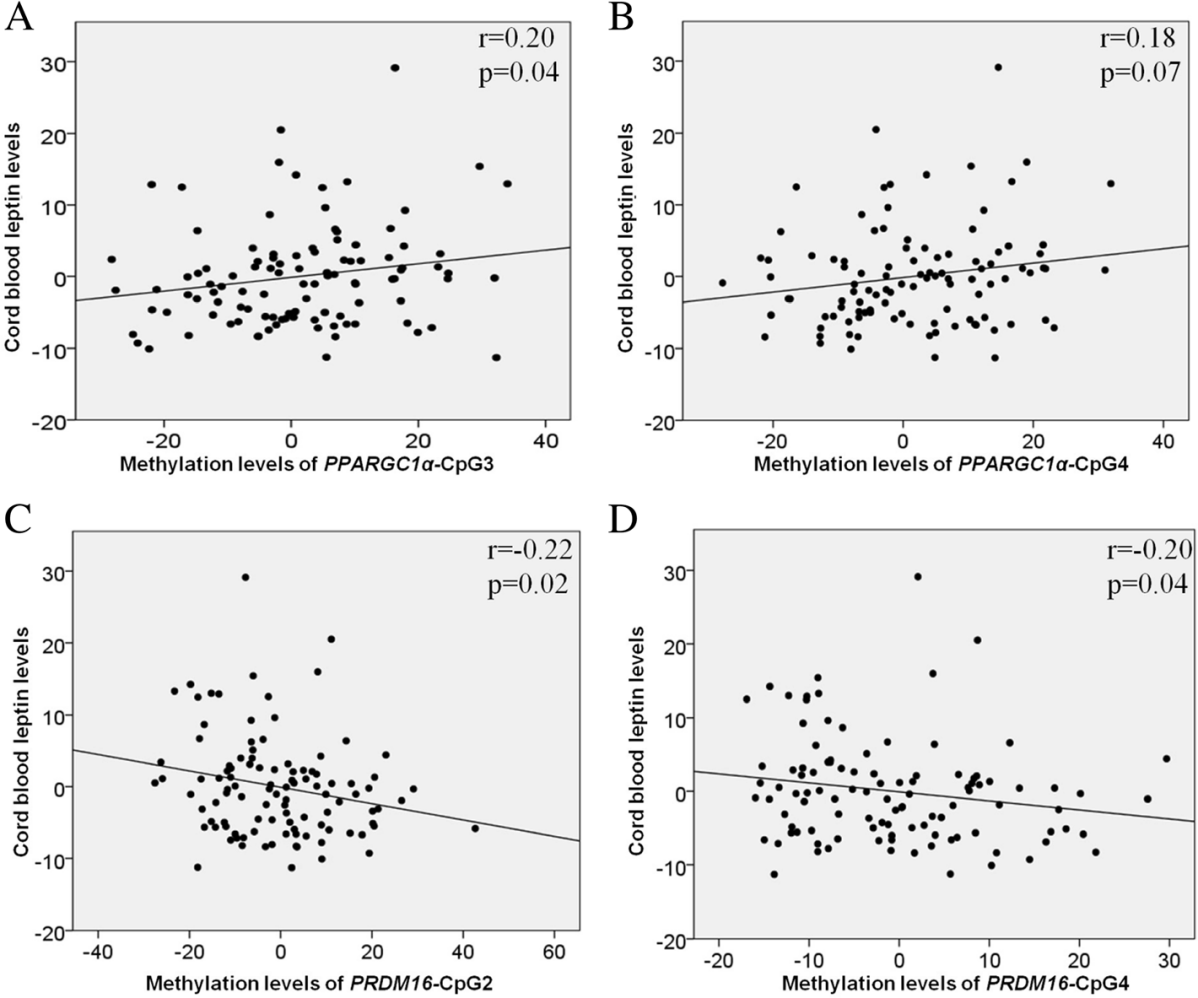
Cord blood leptin levels correlations with DNA methylation. Cord blood leptin levels were correlated with DNA methylation at **a** *PPARGC1α*-CpG3, **b** *PPARGC1α*-CpG4, **c** *PRDM16*-CpG2, and **d** *PRDM16*-CpG4. Unstandardized residuals were first computed for cord blood leptin levels using linear regression and gestational age, newborn’s sex and weight as covariates and for DNA methylation levels using linear regression and gestational age, newborn’s sex and weight, smoking during pregnancy, weight gain between the first and second trimester and maternal BMI at the first trimester as covariates. The residuals were then used to compute Spearman’s correlation coefficients

According to our hypothesis, selected genes are implicated in adipose tissue development. We thus tested associations between epivariations of interest and adiposity markers in newborns. First, we did not observe a significant association between placental DNA methylation levels and birth weight. However, lower DNA methylation levels at *PRDM16* and higher DNA methylation levels at *PPARGC1α* were both correlated with higher cord blood leptin levels (Fig. 2) (Additional file 3: Table S2). The correlation between *PRDM16* DNA methylation and cord blood leptin remained significant after further adjustment for maternal glucose levels at the second trimester of pregnancy, whereas the association between *PPARGC1α* DNA methylation levels and cord blood leptin was modestly reduced and lost significance (CpG3: *r* = 0.20, *p* = 0.04 vs. *r* = 0.17, *p* = 0.097) after adjustment for maternal glycemia. Multivariable linear regression analyses showed that 15 % (*r*^2^; *p* < 0.001) of the cord blood leptin level variability was explained by DNA methylation levels at *PRDM16*-CpG2 (β = −0.20; *p* = 0.04) and *PPARGC1α*-CpG3 (*β* = 0.23; *p* = 0.02) in addition to gestational age (*β* = 0.21; *p* = 0.03) and smoking during pregnancy (*β* = 0.18; *p* = 0.05). We tested the same multivariable correlation model in Gen3G and found that *PPARGC1α* (*β* = 3.52; *p* = 0.05), gestational age (*β* = 2.57; *p* = 0.007) but not *PRDM16* (β = 0.89; *p* = 0.33) nor smoking during pregnancy (*β* = −5.28; *p* = 0.21) contributed significantly to the regression model (data not shown).

Higher DNA methylation levels at *PPARGC1α* were also associated with higher cord blood glucose levels (*r* = 0.25; *p* = 0.04; *n* = 96) in E-21 (Additional file 3: Table S2). This association remained significant after further statistical adjustments for maternal glycemia at any trimester of the pregnancy (data not shown).

In E-21, higher cord blood leptin levels were correlated with higher birth weight (*r* = 0.27; *p* = 0.006) and chest circumference (*r* = 0.23; *p* = 0.03) and were not associated with a newborn’s height (*r* = 0.05; *p* = 0.64) or head circumference (*r* = 0.02; *p* = 0.87).

### Assessment of potential causal links between maternal hyperglycemia, DNA methylation, and fetal metabolic markers

**Fig. 3:**
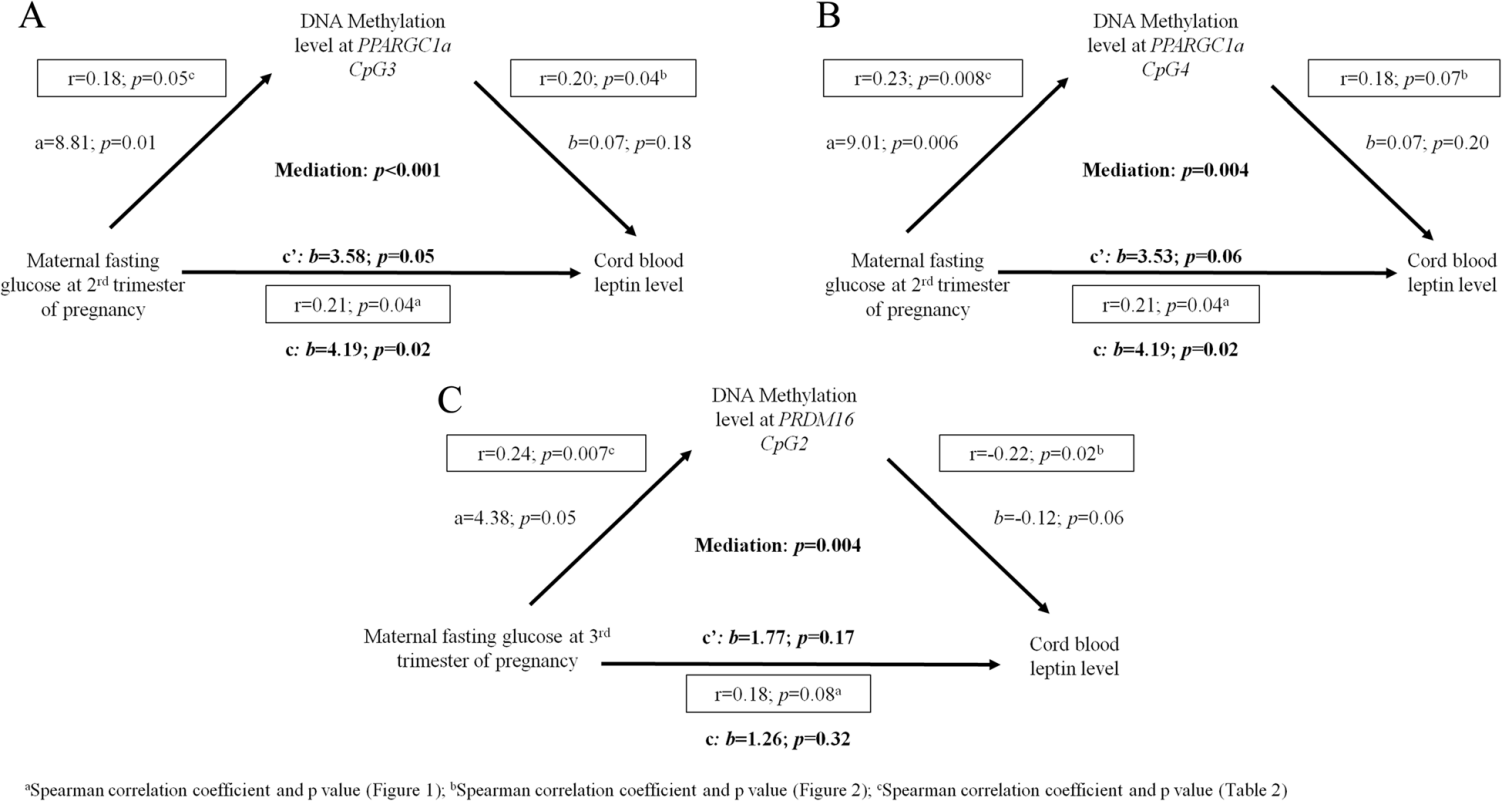
Mediation analyses between maternal glucose levels, DNA methylation variations, and cord blood leptin concentrations. Squared numbers correspond to the coefficients for the total effect with associated *p* values. Path a is the relationship between maternal variables and DNA methylation levels. Path b is the relationship between DNA methylation levels and cord blood leptin levels. Path c is the indirect relationship between maternal variables and cord blood leptin levels, and c‘ is the direct effect of this relationship when taking into account the DNA methylation signature. **a** DNA methylation variations at the *PPARGC1α* - CpG3 gene locus induced by glycemia at the second trimester of pregnancy mediate cord blood leptin levels. **b** DNA methylation variations at the *PPARGC1α* - CpG4 gene locus induced by glycemia at the second trimester of pregnancy mediate cord blood leptin levels. **c** The mediation effect of DNA methylation variations at the *PRDM16* - CpG2 gene locus was demonstrated between maternal glycemia at the third trimester of pregnancy and cord blood leptin levels

Because higher maternal fasting glucose levels were associated with higher cord blood leptin concentrations, we applied mediation analyses to assess whether the potential causal link implicates the DNA methylation variations identified. DNA methylation levels at two CpG sites in *PPARGC1α* significantly mediated the exposure to maternal glycemia at the second trimester of pregnancy and cord blood leptin concentrations (CpG3, *β* = 0.28, *p* < 0.001 and CpG4, *β* = 0.27, *p =* 0.004) (Fig. 3a, b). Our results support that DNA methylation variations at the *PPARGC1α* gene explain 0.8 % of the cord blood leptin level variance independently of maternal glycemia (*p* = 0.05) [17]. Our mediation model might also support that *PRDM16* could mediate the association between maternal hyperglycemia at the third trimester of pregnancy and cord blood leptin levels (CpG2, *β* = 0.14, *p* = 0.004) (Fig. 3c), but the size of the direct effect (correlation between maternal hyperglycemia at the third trimester of pregnancy and cord blood leptin levels—c path) is too small (*p* > 0.05) to support conclusive results.

## Discussion

To the best of our knowledge, this study is the first to show that maternal glycemia is associated with fetal DNA methylation variations in placenta at *PRDM16*, *BMP7*, and *PPARGC1α*, key genes for BAT/wBAT genesis or activation. Interestingly, these epivariations in the fetal placenta were also associated with cord blood leptin levels (*PRDM16* and *PPARGC1α*), suggesting that epigenetic programming at these loci might affect fetal adipose tissue development and fat accretion at the end of pregnancy. We selected cord blood leptin levels considering that our previous results showed that maternal hyperglycemia is closely linked to cord blood leptin levels [45]. In addition, our mediation analyses support that epigenetic regulation of *PPARGC1α* is implicated in the link between maternal hyperglycemia and elevated cord blood leptin levels, considered as a marker for adiposity in offspring.

Our findings at the *PPARGC1α* gene locus are robust, based on consistency of most of the results in two independent cohorts and the significant mediating effect detected with two CpG sites in intron 5. *PPARGC1α* is involved in mitochondrial biogenesis and the adrenergic thermogenic activation of brown/beige adipocytes [46]. Interestingly, a lower *PPARGC1α* DNA methylation was correlated with higher adipose tissue messenger RNA (mRNA) levels [37]; BAT activation has been shown to increase energy expenditure and has been associated with leanness in numerous animal models and humans [18]. Previous studies have also shown higher levels of *PPARGC1α* DNA methylation in samples from type 2 diabetes (T2D) compared to NGT subjects when quantified in human pancreatic islets, muscle, and subcutaneous adipose tissue (SAT) biopsies [36–38, 47]. Moreover, DNA methylation was higher at the *PPARGC1α* gene locus in previously mentioned tissues of young adults born with a low birth weight, also a known risk factor for T2D [38, 47]. These results and ours are thus consistent with a role of *PPARGC1α* in BAT activation defect in obesity and T2D, potentially through increased DNA methylation levels.

More specifically, we report in the current study robust associations between maternal glycemia and *PPARGC1α* placental DNA methylation levels. In concordance with previous results from Xie et al. [48], these results suggest that maternal hyperglycemia might reduce the thermogenic capacity of newborns and thus increase their long-term risk to develop obesity and metabolic diseases through increased DNA methylation levels. In support of this model, we also report a positive correlation between placental *PPARGC1α* DNA methylation and cord blood leptin levels, a well-known marker of adiposity. This is of particular interest since higher *PPARGC1α* DNA methylation variations measured annually in blood between 9 and 14 years of age were found to be predictive of a greater adiposity over this 5-year period [49]. The predictive value of *PPARGC1α* DNA methylation variations will have to be established, but our results provide some additional evidence in support of the fetal epigenetic metabolic programming of obesity. Nevertheless, we cannot exclude that these findings might be related to other PPARGC1α metabolic roles, such as the metabolic regulation of the skeletal muscle [50]. Overall, our results support that *PPARGC1α* DNA methylation is responsive to metabolic variations related to glucose homeostasis during pregnancy, which might affect BAT activation and the development of obesity.

We also report that DNA methylation levels of CpGs in the *PPARGC1α* proximal promoter was associated with maternal insulin and cord blood glucose levels (specific to E-21). Interestingly, these same or very close CpGs were previously associated with T2D diabetes, low birth weight, and dietary fat consumption [36–38, 47]. These results also suggest that, in addition to some CpGs in intron 5, the proximal promoter is of interest and that the targeted CpGs for fetal metabolic programming and *PPARGC1α* gene expression regulation might be different depending on the maternal clinical status (GDM or not) and the phenotype studied. The importance of both proximal promoter and intron 5 regions is supported by ENCODE data, which reports transcription factor binding sites that regulate leptin signaling, for example, at these two loci [51].

On the other hand, PRDM16 and BMP7 are involved and essential in brown/beige adipogenesis, in contrast to PPARGC1α that contribute to BAT activation. Dayeh et al. recently reported in an EWAS that *PRDM16* gene DNA methylation was decreased in human pancreatic islets from patients with T2D [52]. If we hypothesize that these lower DNA methylation levels at *PRDM16* were programmed in utero and were already decreased at birth, our results are concordant since the adverse environment created by maternal hyperglycemia was associated with lower DNA methylation levels at the *PRDM16* gene locus. Similarly, we report that lower *BMP7* DNA methylation levels were associated with the GDM status of the mother, supporting that brown/beige adipogenesis is (epigenetically) affected by glucose metabolism dysregulation in pregnancy. Studies in humans [16, 53, 54] and mice [55, 56] investigated *BMP7* mRNA levels in various tissues and metabolic conditions such as obesity, diabetes, and the metabolic syndrome. These studies showed that *BMP7* mRNA levels are decreased when the energy metabolism is impaired and that *BMP7* activation leads to an increased energy expenditure, thus reducing the metabolic risks [16, 53–56]. In brief, the current results with *PRDM16* and *BMP7* suggest that brown/beige adipogenesis is increased in response to an exposure to maternal hyperglycemia. Assuming that DNA methylation usually represses gene expression, it could have protective effects in offspring.

Among the strengths of our study is the fact that we report results of an association between maternal glycemic traits and DNA methylation at BAT genes in fetal tissues using two independent prospective pregnancy-birth cohorts. Our study also relies on the longitudinal follow-up of mothers throughout pregnancy as well as anthropometric and metabolic markers in newborns, allowing a better characterization of the impact of maternal hyperglycemia on the DNA methylation of offspring and, moreover, on fat mass development. The application of mediation analyses provided some evidence supporting the fetal metabolic programming of obesity through epigenetic variations. Although the correlation and regression coefficients were statistically significant, the variance explained by DNA methylation variations remains small suggesting that other factors, including other DNA methylation marks, contribute to explain the observed phenotypic variability. The identification of those marks should clearly be pursued. Albeit mediation analyses support some causality inference, they will have to be validated in experimental studies. In addition, the functional impact of the epivariations highlighted in this study will have to be investigated. Although we performed gene expression analyses in placenta samples, the mRNA for the selected genes were all undetectable (Ct > 35). Nonetheless, this does not necessarily mean that they were not expressed in the placenta in the course of fetal development nor that the epigenetic marks tested are not functional in other tissues (supported by ENCODE data; https://www.encodeproject.org/). It thus remains unclear whether the DNA methylation variations observed in the placenta reflect those of other newborn tissues and whether these epivariations will be predictive of childhood obesity. We conducted our analyses in placenta tissue because of its fetal origin and implications in fetal metabolic homeostasis. Other tissues such as the adipose tissue and liver are of clear interest, but for ethical reasons, they are not available from live newborns in population-based pediatric populations. Finally, we observed fewer correlations in Gen3G when assessing the correlation between maternal glycemia and outcomes. It might be explained by the exclusion of GDM mothers and consequently an exposure of the fetus to lower levels of maternal glucose transferred by the placenta.

## Conclusions

In conclusion, our study showed that maternal hyperglycemia is associated with placental DNA methylation variations at key genes involved in the genesis and activation of brown/beige adipocytes. We also showed for the first time that *PPARGC1α* DNA methylation levels in placenta likely contribute to mediate the relationship between maternal glycemia and cord blood leptin levels, which could explain why these newborns have an increased risk of obesity and T2D later in life. Since childhood obesity is increasing rapidly worldwide, longitudinal studies are needed to assess whether these epivariations are predictive of obesity and its related metabolic complications and whether they can be reversed by a healthy lifestyle in early life for example.

## Abbreviations

BAT, brown adipose tissue; BMP7, bone morphogenetic protein 7; CTBP2, C-terminal binding protein 2; DOHaD, Developmental Origins of Health and Disease; E-21, Ecogene-21 birth cohort; EWAS, epigenome-wide association study; GDM, gestational diabetes mellitus; Gen3G, Genetics of Glucose regulation in Gestation and Growth; HOMA-IR, homeostasis model assessment of insulin resistance; IADPSG, International Association of the Diabetes and Pregnancy Study Groups; MYF−, myogenic factor 5 negative; MYF+, myogenic factor 5 positive; NGT, normoglycemic; OGTT, oral glucose tolerance test; PPARGC1α, peroxisome proliferator-activated receptor-gamma, co-activator 1, alpha; PRDM16, PR domain-containing protein 16; SAT, subcutaneous adipose tissue; T2D, type 2 diabetes; wBAT, brown-like adipocytes within white adipose tissue; WHO, World Health Organization

## Additional files

Additional file 1: Figure S1. Loci analysed in E-21. *PRDM16* (A), *BMP7* (B), *CTBP2* (C) and *PPARGC1α* (D) genes CpGs epigenotyped are shown. The CpGs within *BMP7*-A. *CTBP2*-A and *PPARGC1α*-A locus were significantly well correlated with each other. For Gen3G, when the CpGs identified in E-21 was covered by 450k array probesets, the exact same CpGs were selected (*cg01046951; ≠cg04873098; ¥cg08550435). Since some CpGs were not covered by the 450k array, probesets covering variable CpGs in close vicinity to those identified in E-21 were selected. *PRDM16*: cg06814194 (1st intron) and cg23738647 (exon 6); *BMP7*: cg18759209 (proximal promoter); and *PPARGC1α*: cg11270806 and cg27514608 (both intron 5). (PDF 180 kb) Additional file 2: Table S1. Assay design for pyrosequencing analysis of the DNA methylation of the *PRDM16*, *BMP7*, *CTBP2* and *PPARGC1α* gene loci. (PDF 180 kb) Additional file 3: Table S2. Spearman’s correlation coefficients between placental DNA methylation and metabolic variables of newborns in E-21 and Gen3G cohorts. (PDF 268 kb)
