# Supplementary material for: PPARGC1α gene DNA methylation variations in human placenta mediate the link between maternal hyperglycemia and leptin levels in newborns
Source: Clin Epigenetics. 2016 Jun 22;8:72. doi: 10.1186/s13148-016-0239-9 (PMC4918074; doi:10.1186/s13148-016-0239-9)
Supplement: Additional file 1: Figure S1. — Loci analysed in E-21. PRDM16 (A), BMP7 (B), CTBP2 (C) and PPARGC1α (D) genes CpGs epigenotyped are shown. The CpGs within BMP7-A. CTBP2-A and PPARGC1α-A locus were significantly well correlated with each other. For Gen3G, when the CpGs identified in E-21 was covered by 450k array probesets, the exact same CpGs were selected (*cg01046951; ≠cg04873098; ¥cg08550435). Since some CpGs were not covered by the 450k array, probesets covering variable CpGs in close vicinity to those identified in E-21 were selected. PRDM16: cg06814194 (1st intron) and cg23738647 (exon 6); BMP7: cg18759209 (proximal promoter); and PPARGC1α: cg11270806 and cg27514608 (both intron 5). (PDF 180 kb) [file 13148_2016_239_MOESM1_ESM.pdf]

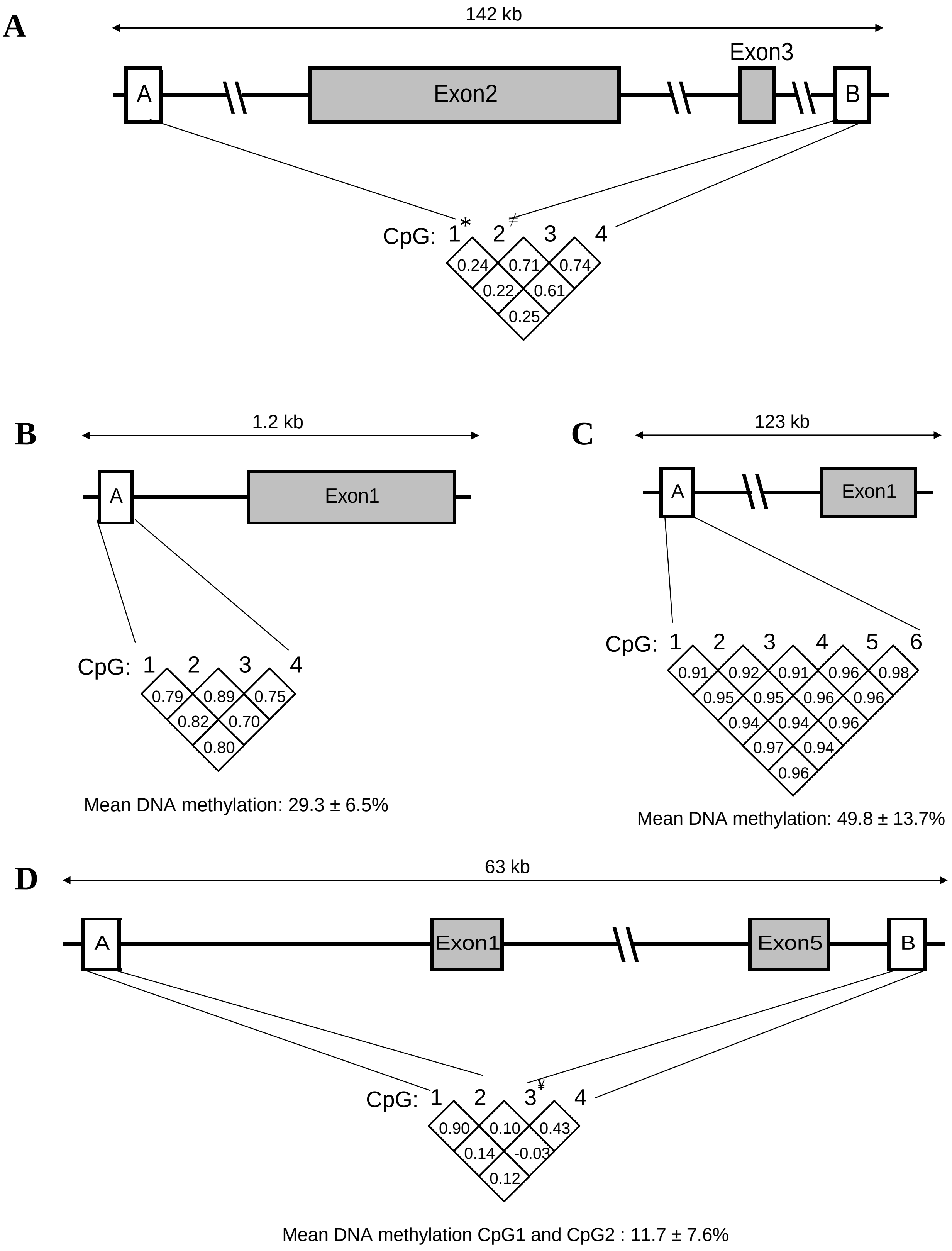
A
142 kb
Exon3
A
Exon2
B
CpG: 1* 2≠ 3 4
0.24
0.71
0.74
0.22
0.61
0.25
B
1.2 kb
A
Exon1
CpG: 1 2 3 4
0.79
0.89
0.75
0.82
0.70
0.80
Mean DNA methylation: 29.3 ± 6.5%
C
123 kb
A
Exon1
CpG: 1 2 3 4 5 6
0.91
0.92
0.91
0.96
0.98
0.95
0.95
0.96
0.96
0.94
0.94
0.96
0.97
0.94
0.96
Mean DNA methylation: 49.8 ± 13.7%
D
63 kb
A
Exon1
Exon5
B
CpG: 1 2 3¥ 4
0.90
0.10
0.43
0.14
-0.03
0.12
Mean DNA methylation CpG1 and CpG2 : 11.7 ± 7.6%

Figure 1S. Loci analysed in E-21. *PRDM16* (A), *BMP7* (B), *CTBP2* (C) and *PPARGC1α* (D) genes CpGs epigenotyped are shown. The CpGs within *BMP7*-A. *CTBP2*-A and *PPARGC1α*-A locus were significantly well correlated with each other. For Gen3G, when the CpGs identified in E-21 was covered by 450k array probesets, the exact same CpGs were selected (*cg01046951; [≠]cg04873098; [¥]cg08550435). Since some CpGs were not covered by the 450k array, probesets covering variable CpGs in close vicinity to those identified in E-21 were selected. P*RDM16*: cg06814194 (1st intron) and cg23738647 (exon 6); *BMP7*: cg18759209 (proximal promoter); and *PPARGC1α*: cg11270806 and cg27514608 (both intron 5).
