# Supplementary material for: PPARGC1α gene DNA methylation variations in human placenta mediate the link between maternal hyperglycemia and leptin levels in newborns
Source: Clin Epigenetics. 2016 Jun 22;8:72. doi: 10.1186/s13148-016-0239-9 (PMC4918074; doi:10.1186/s13148-016-0239-9)
Supplement: Additional file 2: Table S1. — Assay design for pyrosequencing analysis of the DNA methylation of the PRDM16, BMP7, CTBP2 and PPARGC1α gene loci. (PDF 180 kb) [file 13148_2016_239_MOESM2_ESM.pdf]

Supplementary Tables and Figures

Table 1S. Assay design for pyrosequencing analysis of the DNA methylation of the *PRDM16, BMP7, CTBP2* and *PPARGC1α* gene loci

PRDM16-A (1 CpG)	Seq: 5'-CCCTAAAATTTCATCATT-3'<br>F: 5'bio-GTGGTGATAAAAGGTAGAGGAAATAG-3'<br>R: 5'-ACTCAACCCTAAAATTTCATCA-3'	90 bp

gDNA
ACTTAAAAGGATTAAACGGACCTCGGAGGAGCCTCGGCGTTTCACCCGTGGTGACAA
AAGGCAGAGGAAACAGAAGGGAGCACGGCTTTGTCCCCAAATTCCCACTTATAC[1]GA
AATGATGAAATTTCAGGGCTGAGCATCCATCGTGGGCCCCGGGAACCGGCCTCTCAG
CCTCTCTTTTCGCTTCTCTCTAGTCATAAGCATTATCTTGGTCT

Bisulfite treated DNA
ATTTAAAAGGATTAAACGGATTTCGGAGGAGTTTCGGCGTTTTATTCGTGGTGATAA
AAGGTAGAGGAAATAGAAGGGAGTACGGTTTTGTTTTTAAATTTTTATTTATAC[1]GAA
ATGATGAAATTTTAGGGTTGAGTATTTATCGTGGGTTTCGGGAATCGGTTTTTTAGTT
TTTTTTTTCGTTTTTTTTTAGTTATAAGTATTATTTTGGTT

PRDM16-B (3 CpGs)	Seq: 5'-AAAATTTATAAACCCATCA-3'<br>F: 5'bio-AGGTTTAGAGGAGGGTTAAA-3'<br>R: 5'-ACCCAATAAAACAAACCATAATTCTA-3'	238 bp

gDNA
GGGACCTGGAAAAAAAACCCTTCAGAAGCTTCGGAAAAAACGCATCCACCCCAGCC
AGCCCCTAGGGCGAGGGGCGGGAGTGCAGAGGCTTAGAGGAGGGTCAAAAATAAAT
CGAGCTAATAAAGTCATTCC[3]GCC[2]GGAAGAGGGGCC[1]GGTGATGGGTCCATAAATCT
CTCCCGGTTATTATTATTACGTACAAAATGCTACAGGTCATAAAAGTCGTCTGATAGC
CCAGCCTTGCATTGTCCAAACAGGGTGATTGATTGATAATGACGATAATTTGTGAAA
TGATTATTTGGATGTTAGAACCATGGCCTGTCCCACTGGGCTGTTCTGTGGGCGTTTT
CTATAGCCCGGCCCGGGCAAAATGCCTCTGGTAA

Bisulfite treated DNA
GGGATTTGGAAAAAAAATTTTTTAGAAGTTTCGGAAAAAACGTATTTATTTTAGTTA
GTTTTTAGGGCGAGGGGCGGGAGTGTAGAGGTTTAGAGGAGGGTTAAAAATAAATC
GAGTTAATAAAGTTATTTC[3]GTC[2]GGAAGAGGGGTC[1]GGTGATGGGTTTATAAATTTTT
TTCGGTTATTATTATTACGTATAAAATGTTATAGGTTATAAAAGTCGTTTGATAGTTT
AGTTTTGCATTGTTTAAATAGGGTGATTGATTGATAATGACGATAATTTGTGAAATGA
TTATTTGGATGTTAGAATTATGGTTTGTTTTATTGGGTTGTTTTGTGGGCGTTTTTTAT
AGTTCGGTTCGGGTAAAATGTTTTTGGTAA

BMP7 (4 CpGs)	Seq: 5’-GGGATTGAGGGGTAAG-3’<br>F: 5’-GGGGTTAGAGTGGTTGGTTTAAAA-3’<br>R: 5’bio-AAAAAAATCCAAACCCACCCTACCCTCTC-3’	214 bp

The second CpG (C^{2}) contains a listed SNP (rs116320975; G/A).The reported minor allele frequency of the A is less than 0.01%.

gDNA
GCGAGCGCAGAGACAGGCTGGCAACGGCTTCAGGGAGGCGCGGAGGGGTCAGCGTG
GCTGGCTTAAAAAGATACAGGGACTGAGGGGCAAGACC^{1}GGCTCAAGGGTCACC^{2}GC
TTCCAGGAAGCCTTCTATTTCC^{3}GC^{4}GCCACCTCCGCGCTCCCCAACTTTTCCCACCG
CGGTCCGCAGCCCACCCGTCCTGCTCGGGCCGCCTTCCTGGTCCGGACCGCGAGTGC
CGAGAGGGCAGGGCCGGCTCCGATTCCTCCAGCCGCATCCCCGCGACGTCCCGCCAG
GCTCTAGGCACCCCGTGGGCACTCAGTAAACATTT

Bisulfite treated DNA
GCGAGCGTAGAGATAGGTTGGTAACGGTTTTAGGGAGGCGCGGAGGGGTTAGCGTG
GTTGGTTTAAAAAGATATAGGGATTGAGGGGTAAGATC^{1}GGTTTAAGGGTTATC^{2}GTT
TTTAGGAAGTTTTTTATTTTC^{3}GC^{4}GTTATTTTCGCGTTTTTTAATTTTTTTATCGCGG
TTCGTAGTTTATTCGTTTTGTTCGGGTCGTTTTTTGGTTCGGATCGCGAGTGTCGAGA
GGGTAGGGTCGGTTTCGATTTTTTTAGTCGTATTTTCGCGACGTTTCGTTAGGTTTTAG
GTATTTCGTGGGTATTTAGTAAATATTT

CTBP2 (6 CpGs)	Seq: 5’-GGATGGAGGAGATTATAT-3’<br>F: 5’-GGTATTTAGGGATGGAGGAGATTA-3’<br>R: 5’bio-CCAAAACCCCCCAAACTCTT-3’	252 bp

gDNA
GAAAAGGGTCGTGCAGCTCGGTGGCCTCTCCTCCGGCCCCCTGGCCCTGGCTGTAAG
TTTCCTCGGCTCTGCAGGGCGCGGGGCACTCAGGGATGGAGGAGACCACACC^{1}GC^{2}GC
^{3}GGCTGGGTTC^{4}GC^{5}GGAATGGC^{6}GGTCCCTGCGGCCTCCAAAGGCCGGACCTCAGACC
CCTCCCACTCCTTTCCTGCTCGACATCTCAATCCAGCTCCTAATATCTCAATAGGGTT
TTTAAACAAATCGATCCTAAGGGCTCAGTTCAGCCCACCCAGGGATTCTCGGAGGAC
GCACGCCGAACTCAGGGTAGTGAGAAGAGTTTGGGGGGCCCTGGGGCGCAGGGAGA
AGACCCCACACTCCA

Bisulfite treated DNA
GAAAAGGGTCGTGTAGTTCGGTGGTTTTTTTTTCGGTTTTTTGGTTTTGGTTGTAAGTT
TTTTCGGTTTTGTAGGGCGCGGGGTATTTAGGGATGGAGGAGATTATATC^{1}GC^{2}GC^{3}GT
TGGGTTC^{4}GC^{5}GGAATGGC^{6}GGTTTTTGCGGTTTTTAAAGGCCGGATTTTAGATTTTTTT
TATTTTTTTTTTGTTCGATATTTTAATTTAGTTTTTAATATTTTAATAGGGTTTTTAAAT
AAATCGATTTTAAGGGTTTAGTTTAGTTTATTTAGGGATTTTCGGAGGACGTACGTCG
AATTTAGGGTAGTGAGAAGAGTTTGGGGGGTTTTGGGGCGTAGGGAGAAGATTTTAT
ATTTTA

*PPARGC1*α-A (2 CpGs)	Seq: 5’-TTAAGGTAGTTAGGGAGGAAA-3’<br>F: 5’-TTAGGGTATTAGGGTTGGAATTTAATGT-3’<br>R: 5’bio-ACTTCCTTCTAATTATTTCCATTTCTCTC-3’	191 bp

gDNA
CCCCTCAGTTCACAGACATTCTTGATTTCAAAACGCAAACTACACAACCCAGGGCAC
TAGGGTTGGAATTCAATGTTTATTCAAAAAGGCACCCTAAGGCAGTTAGGGAGGAAA
C[1]GCTACATGTATGAAAAATAGGAGCC[2]GGGAATCAAAGCTGATCTGAGCAGAGCAG
CAGCGACTGTATTTACTAACACTTGTTTTCTGGGAGCCTATGAGAGAAATGGAAATA
ATTAGAAGGAAGCTGAAAGGATGGGGTTTTATGGCTTGTTCTCCTTATATGGAGCAA
AGAAAAC

Bisulfite treated DNA
CCCCTCAGTTCACAGACATTCTTGATTTCAAAACGCAAACTACACAACCCAGGGCAC
TAGGGTTGGAATTCAATGTTTATTCAAAAAGGCACCCTAAGGCAGTTAGGGAGGAAA
C[1]GCTACATGTATGAAAAATAGGAGCC[2]GGGAATCAAAGCTGATCTGAGCAGAGCAG
CAGCGACTGTATTTACTAACACTTGTTTTCTGGGAGCCTATGAGAGAAATGGAAATA
ATTAGAAGGAAGCTGAAAGGATGGGGTTTTATGGCTTGTTCTCCTTATATGGAGCAA
AGAAAAC

*PPARGC1*α-B (2 CpGs)	Seq: 5’-TTAATTTTTTGTTTTAGTTTTATTAG-3’<br>F: 5’-AGTTGTTGGTGAGGGTATT-3’<br>R: 5’bio-CTATATCCAAAAAAAAATTTCTTCCC-3’	153 bp

gDNA
CAGTCACAACACTTACAAGGTAGGCTGGGGACCAAAACGAGCTGTTGGTGAGGGCA
CTGGAAGCAAAGAAAGCACATGCAAAGTCTGAGAACACACCAACTTCTTGCCTCCGT
CCCATCAGC[1]GTCACTCTGTGGAAAGAGAATTCAGCACTTATTACAGAGC[2]GGGGGAA
GAAACCTTTCCTTGGACACAGGTTAAACATATTCTATTCAGCTGATAGGCAGTCTAAT
TCACAAGCACCAAAAGACTCCTGAAATGTTAGCCAGGTG

Bisulfite treated DNA
TAGTTATAATATTTATAAGGTAGGTTGGGGATTAAAACGAGTTGTTGGTGAGGGTAT
TGGAAGTAAAGAAAGTATATGTAAAGTTTGAGAATATATTAATTTTTTGTTTTCGTTT
TATTAGC[1]GTTATTTTGTGGAAAGAGAATTTAGTATTTATTATAGAGC[2]GGGGGAAGA
AATTTTTTTTTGGATATAGGTTAAATATATTTTATTTAGTTGATAGGTAGTTTAATTTA
TAAGTATTAAAAGATTTTTGAAATGTTAGTTAGGTG
