# Supplementary material for: PPARGC1α gene DNA methylation variations in human placenta mediate the link between maternal hyperglycemia and leptin levels in newborns
Source: Clin Epigenetics. 2016 Jun 22;8:72. doi: 10.1186/s13148-016-0239-9 (PMC4918074; doi:10.1186/s13148-016-0239-9)
Supplement: Additional file 3: Table S2. — Spearman’s correlation coefficients between placental DNA methylation and metabolic variables of newborns in E-21 and Gen3G cohorts. (PDF 268 kb) [file 13148_2016_239_MOESM3_ESM.pdf]

Table 2S. Spearman's correlation coefficients between placental DNA methylation and metabolic variables of newborns in E-21 and Gen3G cohorts

	E-21 cohort		Gen3G cohort	
**DNA methylation levels[a]**	**Leptin level n=105**	**Glucose level n=74**	**Leptin level n=167**	**Glucose level n=168**
***PRDM16*-CpG1**	r=-0.02<br>NS	r=-0.14<br>NS	r<-0.01<br>NS	r=-0.05<br>NS
***PRDM16*-CpG2**	**r=-0.22**<br>**p=0.02**	*r=-0.19*<br>*p=0.09*	r<-0.01<br>NS	r=0.11<br>NS
***PRDM16*-CpG3**	r=-0.14<br>p=0.14	r=-0.13<br>NS	-<br>-	-<br>-
***PRDM16*-CpG4**	**r=-0.20**<br>**p=0.04**	**r=0.20**<br>**p=0.04**	-<br>-	-<br>-
**Mean *BMP7***	r=-0.11<br>NS	r=-0.06<br>NS	r<-0.01<br>NS	r=-0.04<br>NS
**Mean *CTBP2***	r=-0.16<br>NS	r=-0.11<br>NS	-<br>-	-<br>-
**Mean *PPARGC1α*-CpG1,2**	r=-0.12<br>NS	**r=0.25**<br>**p=0.04**	-<br>-	-<br>-
***PPARGC1α*-CpG3**	**r=0.20**<br>**p=0.04**	*r=0.21*<br>*p=0.06*	r=-0.06<br>NS	r=0.12<br>p=0.12
***PPARGC1α*-CpG4**	*r=0.18*<br>*p=0.07*	r=-0.02<br>NS	-<br>-	-<br>-

[a]Residual scores of DNA methylation levels were used in the statistical models. They were obtained by using unstandardized analysis of residuals computed by linear regressions which included: gestational age, newborn's sex and weight, smoking during pregnancy, weight gain between 1st and 2nd trimester and maternal BMI at 1st trimester. Statistically significant results ($p \leq 0.05$) are shown in bold, whereas statistical trends ($p \leq 0.10$) are shown in italics. NS = $p > 0.15$
